# Addressing the “elephant in the room” of AI clinical decision support through organisation-level regulation

**DOI:** 10.1371/journal.pdig.0000111

**Published:** 2022-09-15

**Authors:** Joe Zhang, Heather Mattie, Haris Shuaib, Tamishta Hensman, James T. Teo, Leo Anthony Celi

**Affiliations:** 1 Institute of Global Health Innovation, Imperial College London, London, United Kingdom; 2 Department of Biostatistics, Harvard T H Chan School of Public Health, Harvard University, Cambridge, Massachusetts, United States of America; 3 Department of Clinical Scientific Computing, Guy’s and St. Thomas’ Hospital NHS Foundation Trust, London, United Kingdom; 4 Department of Critical Care, Guy’s and St. Thomas’ Hospital NHS Foundation Trust, London, United Kingdom; 5 The Australian and New Zealand Intensive Care Society Centre for Outcome and Resource Evaluation, Camberwell, Australia; 6 Department of Neurology, King’s College Hospital NHS Foundation Trust, London, United Kingdom; 7 London Medical Imaging & AI Centre, Guy’s and St. Thomas’ Hospital, London, United Kingdom; 8 Institute for Medical Engineering and Science, Massachusetts Institute of Technology, Cambridge, Massachusetts, United States of America; 9 Division of Pulmonary, Critical Care and Sleep Medicine, Beth Israel Deaconess Medical Center, Boston, Massachusetts, United States of America; Ben-Gurion University of the Negev, ISRAEL

Consider the following proprietary artificial intelligence (AI) algorithm products: (1) continual monitoring to predict likelihood of acute kidney injury (*Dascena Previse*, Dascena, USA); (2) predicting significant events for patients on intensive care (*CLEWICU*, CLEW Medical, Israel); (3) an early warning system for acute inpatient deterioration (*Wave Clinical Platform*, Excel Medical, USA); and (4) using electronic health record (EHR) data to predict likelihood of sepsis (*Epic Sepsis Model*, Epic Systems Corporation, USA).

These algorithms provide early signals of potentially treatable events using real-time clinical data. However, the first three are considered software as a medical device (SaMD) under oversight of the US Food & Drug Administration (FDA) [1–3]. In contrast, the last has undergone no visible regulatory scrutiny [4] and demonstrates minimal data or algorithmic transparency [5], yet is actively used in hundreds of hospitals in the United States that employ the Epic EHR [6]. In 2021, an independent evaluation of this sepsis model demonstrated poor performance (relative to vendor reported metrics), failing to identify 67% of patients with sepsis, with a positive predictive value of 12% and substantial alert burden for clinicians [7]. Other technology vendors [8–10] and healthcare providers [11,12], are also known for hosting development and operationalisation of proprietary algorithmic clinical decision support (CDS). It is likely that many AI implementations fly under the radar.

The elephant then, sitting next to the FDA, is the different consideration given to algorithmic devices for market, and proprietary algorithms developed within existing EHR (traditionally outside of FDA scope [13]). With increasing appearance of CDS, the 21st Century Cures Act 2016 introduced statutory SaMD definitions, such that a non-device CDS is defined by provision of recommendations where clinicians can review the basis for predictions. This could arguably be applied to many algorithms classified as SaMD, and proposed 2019 guidance clarified that CDS must only “recommend” (rather than “drive”) decisions, while creating no intention that “the healthcare provider rely primarily on any of such recommendations to make a clinical diagnosis or treatment decision…” [14]. This distinction remains imprecise. Unlike AI for diagnostic imaging that provides a clear signal (e.g. “there is a nodule”), AI algorithms using EHR data are positioned in complex environments amongst many extraneous considerations; the line between “drive” and “recommend” is consequently blurred, regardless of explainability in underlying intuition, and parallel clinician input is almost always obligatory.

We now observe a resultant dichotomy where the same predictive algorithm might receive different categories of oversight depending on context. This situation poses safety risk:

(1) The FDA considers “recommendation” to pose less risk than decision-making SaMD, but this is arguable. Recommendation flags are an unavoidable additional data-point, and incorrect recommendations may tip decisions towards delayed action or create alert fatigue as much as decision-making SaMD. It is notable that a device for detecting sepsis (*AWARE*, Ambient Clinical Analytics, USA) received FDA classification of moderate-to-high risk (Class II) whereas the Epic sepsis model was deployed without FDA clearance.

(2) AI CDS largely depend on EHR data. By nature, data quality is variable, being dependent on documentation and coding practices. Demographic data such as race-ethnicity may be missing during training and validation. The risk of algorithmic bias is not trivial and cannot be mitigated by clinician “review” of the recommendation.

(3) AI CDS often produce rapid-cycle recommendations on real-time data with dynamic characteristics, introducing need to re-calibrate/re-train algorithms over time. While FDA has introduced lifecycle [15] and adaptive SaMD [16] guidance, these themes of continuous monitoring are equally relevant to unregulated AI CDS.

(4) Clinicians historically use risk scores to guide decisions [17]. In contrast to proprietary EHR CDS, such risk scores are peer-reviewed and when calculated are used situationally. Decisions to employ risk scores in contextually validated and interpretable environments are taken out of clinicians’ hands; deployment is driven, in part, by incentivised system vendors rather than evidence-based guidelines.

(5) Finally, and most importantly—without requirement for oversight, there is no assurance that CDS are accurate in their predictions; no ‘post-market’ evaluation of unintended consequences; and no confidence that risks are suitably handled. EHR vendors cannot simply reassure providers and patients that their opaque, internal procedures to build these algorithms are robust.

The current climate of AI CDS raises patient safety concerns. Based on 2019 FDA non-binding recommendations, moderate-to-high risk, explainable CDS algorithms will likely remain unregulated. The FDA could decide to expand oversight, for example by including all algorithms above a risk threshold. This would be in line with European Union consideration of any Medical Device software which influences therapeutic decisions at a minimum of Class IIa (requiring notified body assessment) [18]. However, for both FDA and EU MDR bodies, the required scalability to handle future volumes of AI CDS is a challenge [19]. But the resulting bottlenecks may stifle innovation, in a period of accelerating AI development [20].

A possible solution is to embrace this dichotomy and regulate according to differences between device manufacturers (who sell focused devices to a wider market), and healthcare provider/ vendor partnerships (who iterate on numerous and diverse CDS for local adoption). Regulators are transitioning to a lifecycle approach for SaMD, with requirements for manufacturers to demonstrate quality management systems across the entire lifecycle, including continuous safety and effectiveness monitoring. This approach should also apply to AI CDS with oversight of the processes employed to create them, rather than the devices.

System views of regulation have been previously discussed [19,21]. In the context of AI CDS, this means defining “AI-ready” organisation/vendor partnerships that can independently deploy AI algorithms onto internal pathways, while maintaining quality and safety. While proposing a detailed framework is outside scope of this piece, any organisation-level approach must consider: (1) maturity of digital infrastructure; (2) functioning relationships with systems suppliers; (3) clear quality systems for evaluation; (4) workforce training and involvement; and (5) transparency in data, development, and outcomes for external audit. These elements are outlined in greater detail in *Table 1.*

**Table 1:** Key components of organisation-level regulation. General good practices that may feed into regulation are laid out in the FDA/MHRA joint principles for Good Machine Learning Practice [22].

Theme	Description
*Infrastructure*	A regulator must ensure that there is sufficient digital maturity within an organisation to safely deploy AI. This includes demonstration of usability within existing digital systems, infrastructure stability with respect to downtime, and data quality and interoperability pre-requisites that are required to support data-driven algorithms.
*Systems supplier relationship*	Safety is reliant on a responsive working relationship between healthcare provider organisation and systems suppliers, to enable rapid response to safety issues, adaptive deployment of software updates, and iteration on front-end and back-end features in response to end-user feedback.
*Quality management systems*	As with SaMD developers, an organisation must demonstrate adequate QMS for each stage of the AI lifecycle, including processes for data management, model training, validation, clinical effectiveness evaluation, and on-going observation and updates.
*Lifecycle transparency*	Regulators must mandate a minimal reporting requirement such that summary characteristics of data (including distributions), algorithms, performance metrics across multiple validation procedures, and real-world impact summaries (including potential safety incidents and near-misses) are available for external review.
*Workforce*	An “AI-ready” workforce is a key component of safe and effective AI CDS deployment. Regulation would ensure a minimum requirement for user training and involvement, and presence of cross-disciplinary expertise, during use-case identification, designing user interface elements, translating recommendations to clinical actions, monitoring and safety reporting, and other processes.

There are multiple downstream benefits. Trust is placed in organisations, and organisation-vendor partnerships, that have pre-existing duties of care to patients. Requirement for end-user input will benefit workforce development, and tighter integration will reduce distance from concepts to deployment. Reducing reliance on duplicative assessment of individual CDS promotes innovation and limits the scalability problem. Requirements for representative data and processes to guarantee calibration to under-represented groups will result in richer data sources, and will share the burden of detecting and mitigating algorithmic bias across local stakeholders [23].

This approach risks shutting out less digitally advanced organisations. To safely deploy AI CDS, a data pipeline in addition to AI expertise that are typically found in well-resourced, academic, networks are required. Smaller providers serving disadvantaged populations may be left behind. Regardless of how CDS is regulated in the future, pooling resources, data, and expertise through broad and inclusive collaborations, is vital to democratise AI benefits.

Regulating organisations is outside the traditional regulatory scope of the US FDA, the European Medicines Agency, or the UK Medicines and Healthcare products Regulatory Agency. Whether through expansion of reach, or delegation to separate (or new) agencies, organisational-level regulation may be the only feasible approach to ensuring quality and safety in the increasing number of AI CDS in EHRs.
